# Targeting human respiratory syncytial virus transcription anti-termination factor M2-1 to inhibit *in vivo* viral replication

**DOI:** 10.1038/srep25806

**Published:** 2016-05-19

**Authors:** B. Bailly, C.-A. Richard, G. Sharma, L. Wang, L. Johansen, J. Cao, V. Pendharkar, D.-C. Sharma, M. Galloux, Y. Wang, R. Cui, G. Zou, P. Guillon, M. von Itzstein, J.-F. Eléouët, R. Altmeyer

**Affiliations:** 1 Institut Pasteur of Shanghai - Chinese Academy of Sciences, Unit of anti-infective research, Shanghai, 200031, P.R. China; 2 Institute for Glycomics, Griffith University, Gold Coast Campus, Gold Coast, QLD 4222, Australia; 3 Shandong University-Helmholtz Institute of Biotechnology, Qingdao, 266101, P.R. China; 4 INRA, Unité de Virologie et Immunologie Moléculaires (UR892), Jouy-en-Josas, 78352, France; 5 CombinatoRx-Singapore, 138667, Singapore; 6 CombinatoRx, Cambridge, MA 02142, USA; 7 Qingdao Municipal Center for Disease Control & Prevention, Qingdao, 266033, P.R. China

## Abstract

Human respiratory syncytial virus (hRSV) is a leading cause of acute lower respiratory tract infection in infants, elderly and immunocompromised individuals. To date, no specific antiviral drug is available to treat or prevent this disease. Here, we report that the Smoothened receptor (Smo) antagonist cyclopamine acts as a potent and selective inhibitor of *in vitro* and *in vivo* hRSV replication. Cyclopamine inhibits hRSV through a novel, Smo-independent mechanism. It specifically impairs the function of the hRSV RNA-dependent RNA polymerase complex notably by reducing expression levels of the viral anti-termination factor M2-1. The relevance of these findings is corroborated by the demonstration that a single R151K mutation in M2-1 is sufficient to confer virus resistance to cyclopamine *in vitro* and that cyclopamine is able to reduce virus titers in a mouse model of hRSV infection. The results of our study open a novel avenue for the development of future therapies against hRSV infection.

Bronchiolitis is a severe lower-respiratory tract infectious disease primarily caused by members of the *Paramyxoviridae* family. Human respiratory syncytial virus (hRSV) is the principal cause of morbidity in children less than 2 years of age^1^,^2^ as well as the elderly, immunocompromised and transplant patients^3^,^4^,^5^,^6^,^7^. To date, there are neither vaccines nor approved small molecule drugs available to prevent or treat hRSV infection. The immuno-prophylactic antibody palivizumab^8^ is approved for high-risk patients only such as premature babies and infants suffering from underlying diseases^8^,^9^. The broad-spectrum small molecule antiviral ribavirin is available to treat infection, but it has considerable side-effects and limited efficacy^10^,^11^. During the past decade, a number of drug candidates targeting hRSV entry^12^,^13^,^14^,^15^,^16^ or replication steps^17^,^18^,^19^ have been advanced to pre-clinical or clinical development.

The hRSV genomic RNA (vRNA) is packaged by the viral nucleoprotein (N) at all times, forming a N:RNA complex, called nucleocapsid. This ribonucleoprotein complex is used as a template for mRNA transcription and genomic or antigenomic RNA replication by the RNA-dependent RNA polymerase (RdRp), which is composed of 2 major viral proteins: the phosphoprotein P and the large polymerase L^20^. In this complex, the phosphoprotein is an essential co-factor of the L polymerase by binding to L and N and targeting the polymerase L to vRNA^21^,^22^,^23^. Two co-factors, M2-1 and M2-2, are required for the RdRp to process RNA efficiently during the viral cycle. M2-1 is a tetrameric transcription processivity factor that binds in a competitive manner to RNA and P via its core domain^21^,^22^,^24^. M2-1 functions as an anti-terminator of transcription that prevents premature termination of transcription both intra- and inter-genetically^20^,^25^. Although *in vitro* experiments have shown that M2-1 binds preferentially to positive-sense viral gene end (GE) and poly-A sequences^21^,^26^, the exact mechanisms by which M2-1 improves transcription efficiency is not fully understood.

By screening libraries of known bioactive compounds, we identified cyclopamine (CPM) and jervine as highly potent and selective inhibitors of hRSV replication *in vitro*. CPM is a well-known antagonist of the smoothened protein (Smo), a 7-transmembrane receptor of the Sonic hedgehog signaling pathway (Shhp)^27^,^28^ involved in embryonic development, cell differentiation and tumorigenesis^29^. The discovery of the CPM anti-proliferative activity has led to the development of novel, CPM-competitive Smo antagonists for cancer therapy including GDC-0449 (vismodegib), approved for the treatment of basal-cell carcinoma^30^, and LY2940680^31^.

The present report shows that CPM is a potent inhibitor of hRSV infection. Inhibition is not mediated by the cognate Smo receptor, but through the hRSV viral processivity factor M2-1. The CPM-mediated suppression of hRSV infection could also be demonstrated *in vivo*, where hRSV lung titers could be significantly reduced in CPM-treated mice. This novel off-target effect of CPM paves the way to the future development of therapies against hRSV infection, using CPM analogues that target hRSV replication complex, in particular M2-1.

## Results

### Identification of Veratrum steroidal alkaloids as inhibitors of hRSV infection *in vitro*

**Figure 1:**
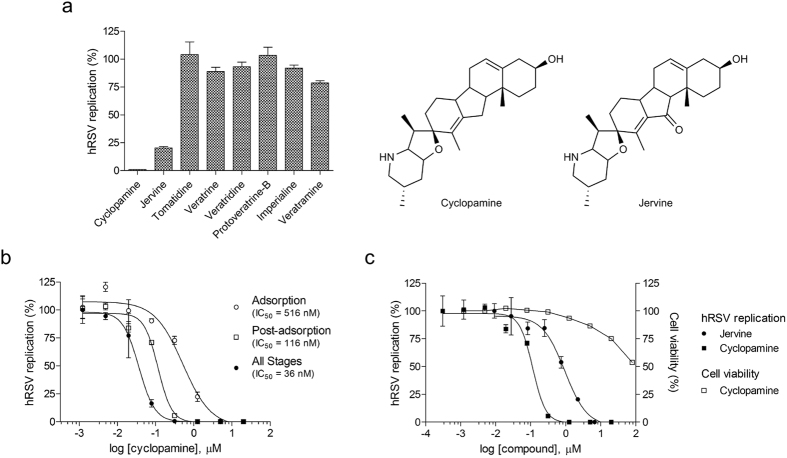
Inhibition of hRSV infection by CPM and jervine *in vitro*. (**a**) Single dose inhibition of hRSV infection of HEp-2 cells by analogues of CPM tested at 3.5 μg/mL (cyclopamine: 8.5 μM, jervine: 8.2 μM, tomatidine: 7.7 μM, veratrine: 5.9 μM, veratridine: 5.2 μM, protoveratrine B: 4.3 μM, imperialine: 8.1 μM, veratramine: 8.5 μM). **(b)** Dose-dependent inhibition of hRSV replication in HEp-2 cells by CPM when present during viral adsorption stage, post-adsorption or at all stages of infection. Antiviral activities were measured by focus reduction assay. Foci numbers (adsorption stage) and sizes (post-adsorption and all stages) were measured automatically using the software FiJi^57^, and were normalized as percentage of the untreated control. **(c)** Dose-dependent inhibition of hRSV replication in HEp-2 cells by CPM and jervine at post-adsorption stage, and cytotoxicity of CPM towards HEp-2 cells. All infections were carried out for 72 h. The cytotoxicity of CPM was measured over 72 h of incubation with cells, by cell viability assay. Each data point represents the mean of duplicate values, and the SEM is represented by the error bars. The graphs and the IC_50_ and IC_95_ values were created and calculated using the software GraphPad Prism v.5 (GraphPad Software, La Jolla California, USA), and are representative of 3 independent experiments.

We performed phenotypic, cell-viability-based screens designed to measure the antiviral effect of compounds on hRSV replication in HEp-2 cells. We screened a 502 compound natural product library and a 3,244 compound approved drug and probe-compound library (hit rates 1.85% and 2.39%, respectively). They allowed us to identify jervine and CPM, two *Veratrum* steroidal alkaloids, as potent *in vitro* anti-hRSV molecules. Other compounds of this class such as veratrine, portoveratrine-B, imperialine or veratramine were inactive against hRSV, indicating a specificity of action of jervine and CPM (Fig. 1a).

A focus reduction assay was used to further characterize the 50%, 90%, 95% or 99% inhibitory concentrations of the compounds (IC_50_, IC_90_, IC_95_ and IC_99_, respectively). CPM (11-deoxojervine) was identified as the most potent inhibitor of hRSV infection with an IC_50_ of 36 nM, IC_90_ of 151 nM, IC_99_ of 336 nM and a selectivity index >2000 when present throughout infection (Fig. 1b). The concentration of CPM resulting in a 50% decrease of cell viability (CC_50_), was found to be >80 μM (Fig. 1c). CPM remained the most active against hRSV when added exclusively post-adsorption as demonstrated by an IC_50_ of 116 nM and IC_90_ of 404 nM (Fig. 1b). In contrast, the potency of CPM was decreased when present exclusively during virus adsorption (IC_50_ 516 nM, IC_90_ 5.4 μM). Jervine also inhibited hRSV replication at post-adsorption stage with higher IC_50_ and IC_95_ values of 994 nM and 6.9 μM, respectively (Fig. 1c). These results suggest that CPM and jervine likely act on a post-entry mechanism of hRSV replication. Due to its greater potency and to the wealth of available literature data on its biological activity, CPM was then used for all further studies.

### CPM inhibits hRSV infection independently of the Smo receptor

**Figure 2:**
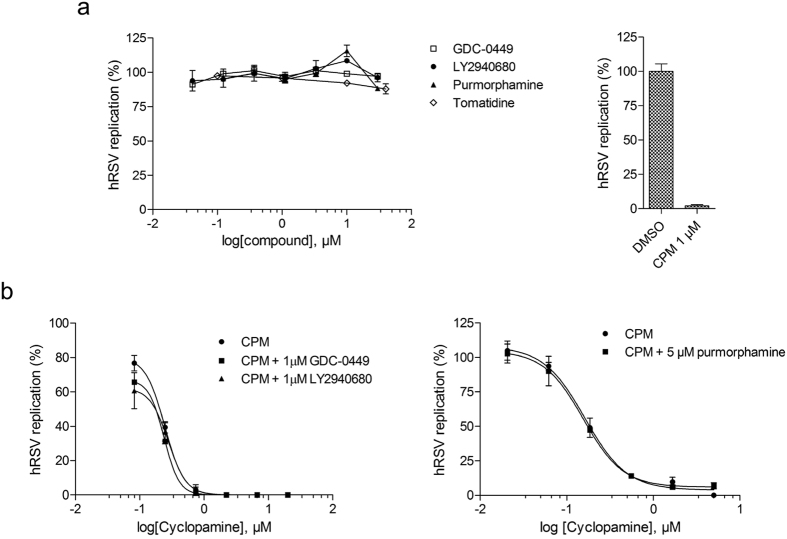
Effect of Smo modulators and a CPM analog on hRSV infection *in vitro*. (**a**) Dose-dependent inhibition of hRSV replication in HEp-2 cells of Smo inhibitors (GDC-0449, LY2940680), an Smo activator (Purmorphamine) and a CPM structural analog (Tomatidine) (left), versus the effect of 1 μM of CPM (right). (**b**) Comparative effect of increasing concentrations of CPM in combination with 1 μM of GDC-0449, LY294068 (left), or 5 μM of purmorphamine (right) on hRSV replication in HEp-2 cells. Antiviral activities in (**a**,**b**) were measured by focus reduction assay, with compounds applied post-adsorption for 72 h. The average size of foci was measured using the software Fiji^57^, and normalized as percentage of the untreated control. Each data point represents the mean of duplicate values, and the SEM is represented by error bars. The graphs were created using the software GraphPad Prism v.5 (GraphPad Software, La Jolla California USA), and are representative of at least 2 independent experiments.

To assess whether Smo is involved in CPM-mediated inhibition of hRSV replication, two Shhp antagonists (GDC-0449, LY2940680) and one agonist (purmorphamine), as well as one non Smo-binding structural analogue of CPM (tomatidine) were evaluated on hRSV infection *in vitro*. As shown in Fig. 2a, all compounds except CPM failed to inhibit hRSV replication at concentrations up to 30 μM. Since GDC-0449, LY2940680 and purmorphamine are known competitors of CPM for binding to Smo^32^,^33^,^34^, we performed a competition assay with increasing concentrations of CPM in presence of the compounds. None of the competitors induced a shift in IC_50_ for CPM-mediated hRSV inhibition (Fig. 2b), leading us to the conclusion that the inhibition of hRSV by CPM is not mediated by its binding to Smo but through a novel, Shhp-independent mechanism.

### The R151K mutation in M2-1 confers resistance to CPM

**Figure 3:**
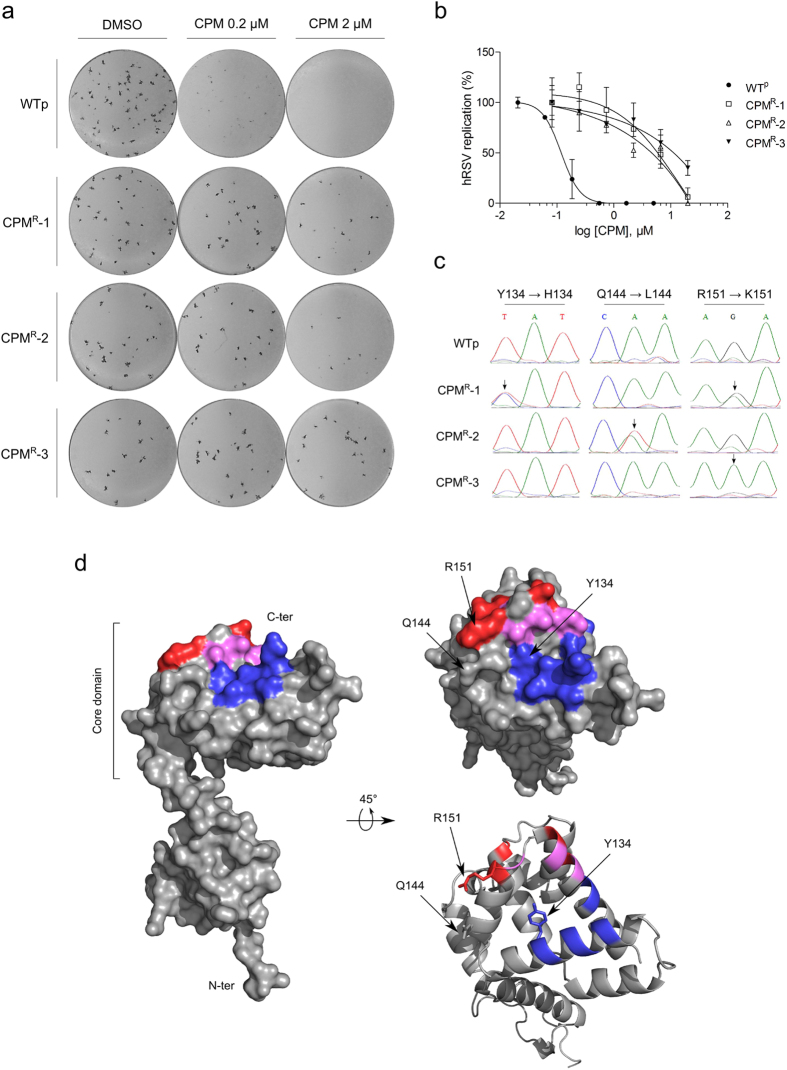
hRSV mutants resistant to CPM. (**a**) Comparison of the focus formation by CPM-resistant viruses CPM^R^-1, CPM^R^-2, CPM^R^-3, and a passaged-wild-type virus (WTp) on HEp-2 cells in the presence or absence of CPM. **(b)** Dose-response of CPM on the 3 resistant mutants and WTp, measured by focus reduction assay on HEp-2 cells. The compound in **(a,b)** was applied post-adsorption, for 72 h. The average size of foci, measured using the software Fiji^57^, was normalized as percentage of the untreated control. The mean of duplicated values are plotted, along with the SEM represented by the error bars. The results are representative of 3 independent experiments. **(c**) Sequencing chromatograms of WTp and the 3 resistant virus supernatants, showing the base changes and amino-acid substitutions. The black arrows indicate the sites of clear mutation. **(d**) Mapping of the three mutation sites on a monomeric M2-1. Left: Surface representation of a full-length M2-1. Right: Surface (top) and cartoon (bottom) representations of the C-terminal side of M2-1 core domain. The RNA and P binding sites described by Blondot *et al.*^21^ are colored in red and blue respectively. The amino-acids that belong to both binding sites are colored in magenta. The three sites of mutation are indicated with black arrows. The models (PDB accession code 4C3B) were rendered using the PyMOL Molecular Graphics System, Version 1.7.1.0, Schrödinger, LLC.

To determine whether a particular viral protein was implicated in the inhibition of hRSV by CPM, we selected CPM-resistant mutants of hRSV by 8 sequential passages in presence of increasing CPM concentrations, from 100 nM to 1 μM. Three CPM-resistant hRSV culture supernatants were generated independently, CPM^R^-1, CPM^R^-2 and CPM^R^-3. They possessed growth characteristics comparable to a wild-type virus passaged in parallel (WTp) and displayed similar focus morphology in the presence and absence of CPM (Fig. 3a). Interestingly, the virus was unable to regain fitness and induce CPE if the concentration of cyclopamine was increased too quickly (i.e. from 400 nM to 800 nM).

The potency of CPM, added post-adsorption, was evaluated for each of the CPM-resistant supernatants. As shown in Fig. 3b, the WTp supernatant was CPM-sensitive with an IC_50_ value of 119 nM, comparable to the original WT hRSV. However, the potency of CPM in resistant virus supernatants was decreased 38- to 96-fold with IC_50_ values of 6 μM, 4.5 μM and 11.22 μM for CPM^R^-1, CPM^R^-2 and CPM^R^-3, respectively, confirming the presence of resistant virus in the culture supernatants.

Genome sequencing and alignment of the supernatants revealed three coding mutations located in the hRSV M2-1 gene (Fig. 3c). While WTp was found to be identical to the original hRSV sequence, CPM^R^-1 and CPM^R^-2 consisted of a mixed population of wild-type and M2-1 variants containing Y134H/R151K and Q144L/R151K mutations, respectively. In contrast, CPM^R^-3 was a homogeneous population displaying only the M2-1 R151K mutation. Interestingly, CPM^R^-3 showed the highest resistance to CPM as measured by a 96-fold increase of the IC_50_. Although the three mutated residues are located within the core domain of M2-1, R151 is located in the α-helix α8, a region previously described for its role in RNA and P binding^21^,^22^ (Fig. 3d). Together, these data demonstrate that the R151K mutation is sufficient to confer resistance to CPM, suggesting that the function of M2-1 as part of the RdRp complex is affected by the compound.

### CPM inhibits M2-1-dependent viral transcription

**Figure 4:**
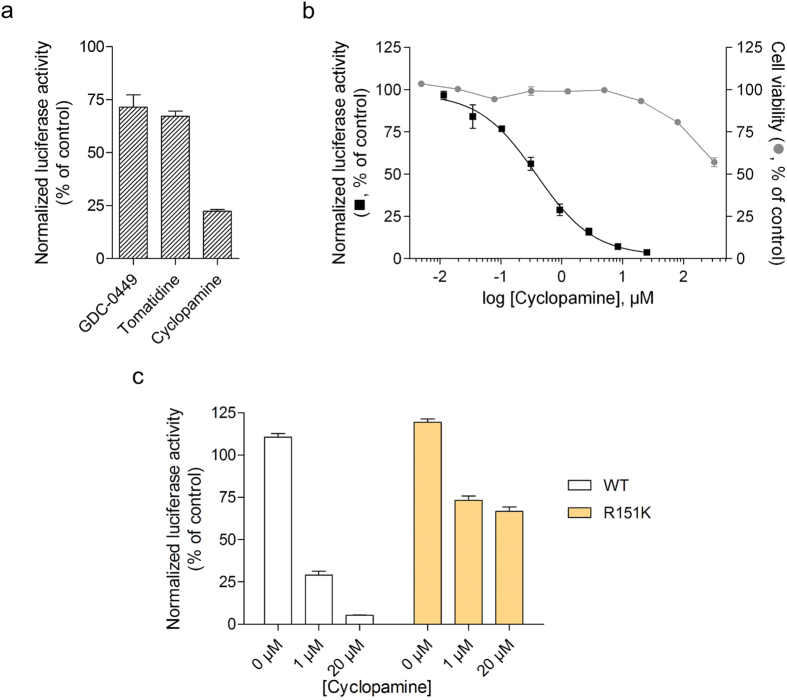
CPM inhibition of hRSV M2-1-dependent transcription. Inhibition of hRSV RdRp by 8.3 μM of CPM, GDC-0449, tomatidine **(a)** and dilutions of CPM **(b)**, measured by RdRp-specific minigenome assay in BSR-T7/5 cells. The cytotoxicity of CPM towards BSR-T7/5 cells is shown in **(b)**. **(c)** Inhibition of hRSV replication complex by CPM measured by minigenome assay in BSR-T7/5 cells transfected with plasmids coding for a WT or a R151K variant M2-1. Viral RNA synthesis in **(a–c)** was quantified by measuring the luciferase activity after cell lysis 24 h after transfection. The error bars represent the standard error of the mean of luciferase activity measured in triplicates, normalized against β-galactosidase activity and expressed as percentage of a control treated with PBS. For the data points at 0 μM, DMSO was used in place of CPM. The results are representative of 2 independent experiments performed in triplicate.

To confirm that the M2-1 mutations were sufficient to confer hRSV resistance to CPM, we used a well-established hRSV-specific minigenome assay system^22^,^26^. It contains the authentic M/SH gene junction, and the Luc reporter gene downstream of the gene start sequence present in this gene junction. The expression of the Luc gene in this system is absolutely dependent on the presence of a functional M2-1^22^. The pM/Luc plasmid was co-transfected in BHK-21 BSR-T7/5 cells expressing T7 RNA polymerase together with p-β-gal, pL, pP, PN, and pM2-1 (WT or mutant). Luciferase activities were determined and normalized based on β-galactosidase expression. The effect of CPM on viral transcription was analyzed using either M2-1^WT^ or M2-1^R151K^. Figure 4a,b shows that CPM, unlike GDC-0449 and tomatidine, inhibits the function of the hRSV RdRp complex in a dose-dependent manner with an IC_50_ of 380 nM while showing low toxicity towards BSR-T7/5 cells at working concentrations (CC_50_ > 320 μM, Fig. 4b). However, in cells expressing M2-1^R151K^ the RdRp activity was highly resistant to CPM with a maximum inhibition of 26% at 1 μM, which could not be significantly increased using higher CPM concentrations (Fig. 4c). This result further validates that CPM blocks the RdRp complex by specifically inhibiting M2-1-mediated hRSV transcription.

### CPM reduces M2-1 expression levels

**Figure 5:**
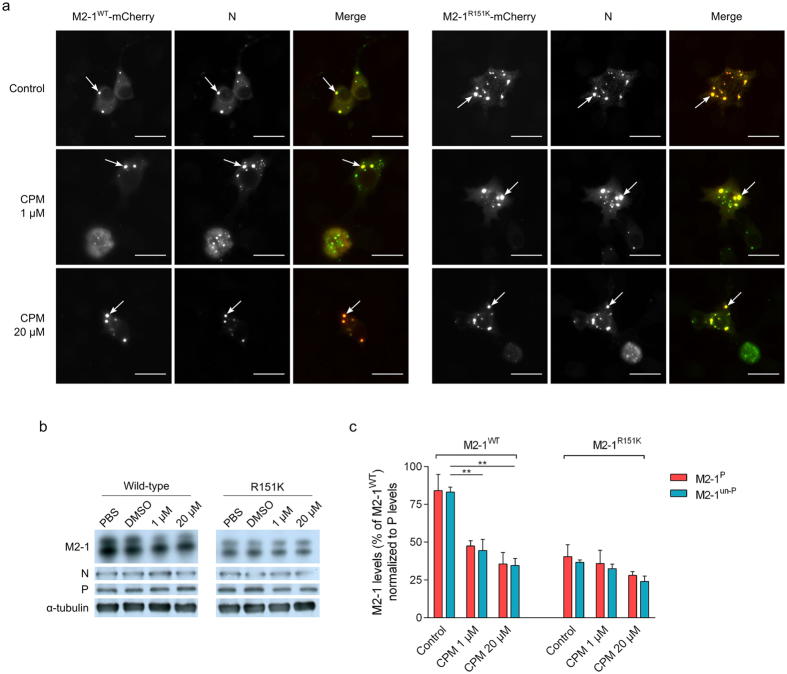
Effect of CPM on M2-1 expression. **(a)** Immuno-fluorescence labelling of N and direct fluorescence of M2-1^WT^-mCherry or M2-1^R151K^-mCherry in minigenome-transfected BSR-T7/5 cells. Transfected cells were treated for 24 h with either DMSO or the indicated concentrations of CPM. N (green) was visualized using a mouse monoclonal anti-N primary antibody. Scale bars: 20 μm. The white arrows point to the location of cytoplasmic inclusion bodies. Merged images were created using the software Fiji. **(b)** Expression of M2-1, P, N and α-tubulin in minigenome-transfected BSR-T7/5 cells with the indicated concentrations of CPM, using M2-1^WT^ or M2-1^R151K^, resolved by SDS-PAGE 24 h post-transfection and revealed by Western-blot using rabbit polyclonal anti-M2-1, P and N and mouse monoclonal anti-α-tubulin antibodies. Levels of α-tubulin are shown as a control of protein expression and DMSO served as control. The results are representative of 3 independent experiments **(c)** Associated levels of phosphorylated (M2-1^P^) and unphosphorylated (M2-1^un-P^) M2-1^WT^ or M2-1^R151K^, normalized to levels of P in the absence of CPM or DMSO, and expressed as a percentage of M2-1^WT^ in the absence of CPM. Proteins levels were measured from western blots using a GeneGnome Digital Imager (Syngene, Frederick, MD, USA). Bars are the mean of 3 independent experiments ± SEM. **p < 0.01 (Two-tailed t-test).

We performed immunofluorescence microscopy on minigenome-transfected cells to determine if CPM had an effect on M2-1 protein expression and subcellular localization. As shown in Fig. 5a, M2-1^WT^ and M2-1^R151K^ were readily detected in inclusion bodies together with N in the presence of increasing concentrations of compound. Overall expression levels of M2-1^WT^, however, were reduced by CPM while levels of N and P remained unaffected (Fig. 5b). Figure 5c shows that both the phosphorylated (M2-1^P^) and unphosphorylated (M2-1^un-P^) forms of M2-1 were reduced by CPM in a dose-dependent manner. Expression levels of M2-1^R151K^ were lower compared to M2-1^WT^, but were not significantly affected by CPM treatment (Fig. 5b,c). These results show that treatment of cells with CPM is correlated with a selective reduction of M2-1 protein levels, and that M2-1^R151K^ is resistant to CPM-mediated down-expression.

### Antiviral efficacy of CPM in a mouse model of hRSV infection

**Figure 6:**
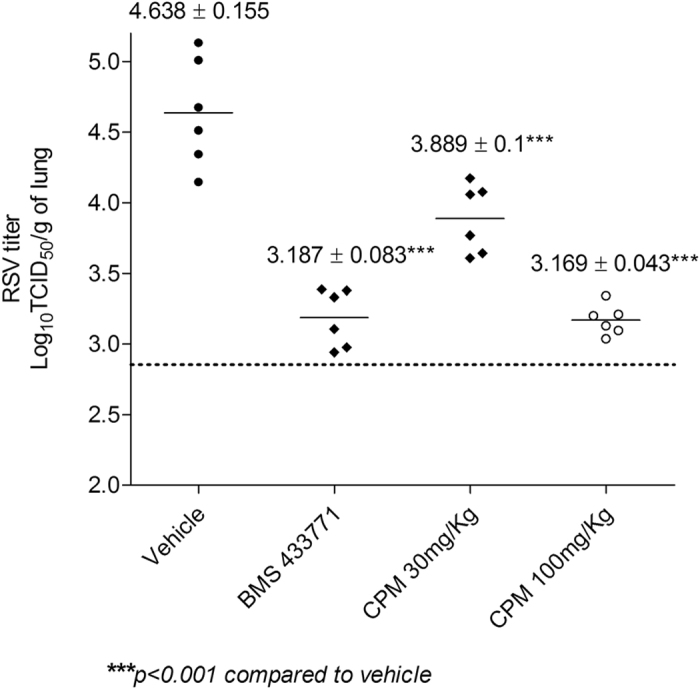
Efficacy of CPM against hRSV in the mouse BALB/c host model of infection. Animals were inoculated intranasally with hRSV Long strain. CPM and BMS-4337714 were administered intraperitoneally and by oral gavage, respectively, as a 4-day b.i.d. regimen in which the first dose was given 1 h before virus inoculation. Treatment cohorts are shown on the abscissa; animals of the infection control group were inoculated with virus and treated with vehicle only. The infectious hRSV lung titers are shown on the ordinate as log_10_ TCID_50_ per gram of lung. Each data point represents the hRSV titer for each individual animal of the respective treatment cohort. The horizontal line, drawn in each cohort, marks the geometric mean hRSV titer of the group, with the corresponding viral titer ± SD stated above. The horizontal hatched line, across the graph, represents the hRSV titer at the limit of assay detection. N = 6 mice per group.

Bioavailability and tissue distribution of CPM, CPM-analogues and Smo antagonists have been widely documented^35^,^36^. We therefore tested if the *in vitro* anti-hRSV activity of CPM could be observed in an experimental mouse model of hRSV infection. CPM was able to reduce lung hRSV titers by 1.5 logs when administered at 100 mg/kg for four days post infection (Fig. 6). The lung titer reduction was statistically significant (p > 0.001) and comparable to that observed with the hRSV fusion inhibitor BMS-477331^15^,^37^ at 50 mg/kg. Importantly, the magnitude of infection inhibition in mice was dose dependent. The animal data extend our *in vitro* observations and suggest that CPM and CPM analogues targeting M2-1 may be a promising avenue for the development of targeted hRSV-specific therapy.

## Discussion

We identified the known Smo antagonist CPM as a potent post-entry inhibitor of hRSV replication by phenotypic screening of compound libraries. Several *Veratrum* steroidal alkaloids were evaluated *in vitro* to assess the structure-activity relationship of CPM. Only the Smo antagonist jervine^38^, a close structural analogue of CPM that possesses a keto-group in C-11 position on the D-ring, was able to inhibit viral replication. This keto-group leads to a 30-fold reduction of potency, providing important clues for future structure-activity relationship (SAR) using CPM analogues. In view of these results, it is also expected that the potent cyclopamine analogue IPI-926 (saridegib)^39^,^40^ would have a similar level of inhibition towards hRSV infection, which could add value to further SAR studies.

We hypothesized that the anti-hRSV activity of CPM may be mediated by Shhp and the Smo receptor. CPM binds to the same domain of Smo as antagonists GDC-0449 (vismodegib, Erivedge^®^) and LY2940680, currently in clinical trials, as well as the Smo-agonist purmorphamine. The 3 compounds, known competitors of CPM for binding to Smo^27^,^32^,^34^,^41^,^42^, had no effect on infection *in vitro* neither individually nor in combination with CPM, strongly suggesting that Smo is not involved in CPM-mediated inhibition of hRSV replication. Interestingly, an anti-Sonic hedgehog antibody was previously shown to reduce hepatitis C virus replication^43^. We did not observe any effect of this antibody on hRSV replication (data not shown), corroborating our conclusion that CPM inhibition of hRSV was independent of Smo and the Shhp.

To characterize the mechanism of action of CPM against hRSV, we selected CPM-resistant viruses and identified that a single R151K mutation on the hRSV M2-1 protein was able to confer resistance to the compound. Our data are consistent with previous studies which demonstrated that this residue is critical for RdRp activity. Indeed, an R151D substitution in M2-1 resulted in 70% reduction of M2-1-dependent transcription^21^,^26^. In an hRSV RdRp-specific minigenome functional assay CPM potently inhibited RdRp activity. The R151K variant was significantly less sensitive to CPM, demonstrating that the R151K mutation is responsible for CPM resistance. An analysis of 486 wild-type sequences reported in GenBank showed that R151 is fully conserved in all human M2-1 sequences (data not shown). Furthermore, virus supernatants were unable to recover from a sharp increase of cyclopamine concentration. This strongly suggests that pre-existing resistant R151K mutant quasispecies are unlikely to circulate in significant proportion in the population, and that they are unlikely to recover from a CPM treatment. The role of M2-1 in CPM-mediated hRSV inhibition is also consistent with our observation that CPM had no effect on hPIV-3 replication, which does not encode an M2-1-like protein (data not shown).

CPM significantly reduced M2-1 expression levels in minigenome-transfected cells expressing M2-1^WT^, while M2-1^R151K^ was unaffected. Both phosphorylated and non-phosphorylated forms of M2-1^WT^ were significantly reduced by CPM, in a dose-dependent manner compared to M2-1^R151K^. The overall levels of M2-1^R151K^, however, were lower than the ones of M2-1^WT^. This could be due to a poorer stability of the mutant protein, or a lower expression resulting from the mutation. Nevertheless, since the R151K variant had a phenotype similar to that of the WTp virus, it is possible that CPM uses an additional mechanism other than a reduction of M2-1 levels alone to inhibit viral replication. Although we could not demonstrate a direct effect of CPM on M2-1:P or M2-1:RNA interactions *in vitro* using recombinant proteins (data not shown), we cannot exclude that the reduction of M2-1 levels could be due to a specific effect of CPM on M2-1 binding to specific RNA and/or P, resulting in exclusion of M2-1 from inclusion bodies and protein degradation. By analogy with rhabdoviruses, inclusion bodies are considered as sanctuaries where viral RNA synthesis occurs^44^,^45^. Thus, although M2-1^WT^ and M2-1^R151K^ could still be detected in inclusion bodies in the presence of CPM (Fig. 5a), the levels of M2-1^WT^ (but not M2-1^R151K^) were strongly reduced as compared to untreated cells (Fig. 5c). The overall reduction in M2-1 expression levels induced by CPM is expected to result in reduced viral RNA transcription and consequently virus production. Determining the exact molecular mechanism by which CPM interferes with the M2-1 function in hRSV transcription will require further investigation.

We showed that CPM is able to reduce lung hRSV titers by 1.5 logs in the BALB/c model of infection. Several small compounds inhibiting hRSV infection are currently under development, some of them targeting proteins of the hRSV RdRp complex such as L or N^18^,^19^,^46^,^47^,^48^. Other compounds inhibiting the critical F protein-mediated viral fusion have reached clinical development: the pyrazolopyrimidine GS-5806 has shown efficacy in phase 2a clinical trials^13^ while the azabenzimidazole BMS-433771, when administered orally in the BALB/c mouse model, was able to reduce lung titers of hRSV by 1.5 logs^37^. BMS-433771 efficacy in mice was predictive of efficacy in cotton rats despite different pharmacokinetic and pharmacodynamics properties between the models^15^.

Mousseau *et al.*^49^ have previously reported the discovery of a chemical analogue of the naturally occurring steroidal alkaloid cortistatin A as an inhibitor of human immunodeficiency virus Tat-dependent viral genome replication. Other studies have reported the discovery of inhibitors of the hRSV RdRp complex using small molecule libraries and hRSV minireplicon systems^46^,^47^,^48^,^50^. Compounds have also been used to target the hRSV M2-1 zinc-binding domain, but show limited *in vivo* efficacy and their mode of action remains unclear^51^,^52^.

To our knowledge, our study is the first to report a natural steroidal alkaloid as a specifically targeted antiviral compound showing *in vivo* efficacy. Our results also demonstrate that M2-1 constitutes a promising target for inhibitors of hRSV replication, and pave the way for new approaches in the design of antiviral compounds against hRSV.

## Materials and Methods

### Cells and viruses

Human respiratory syncytial virus Long strain (ATCC reference VR-26) and HEp-2 cells (ATCC reference CCL-23) were used for infection assays. Cells were maintained in DMEM, supplemented with penicillin/streptomycin and 10% FBS. Virus was passaged in HEp-2 cells in similar conditions, with 2% FBS. Virus stocks were prepared using HEp-2 cells, at low multiplicity of infection (MOI) for two to three days. Cells and virus-containing supernatant were then subjected to a single freeze/thaw cycle to release cell-bound virus, and samples were clarified by centrifugation at 2000 × g for 10 min at 4 °C. The supernatants were homogenized, aliquoted and stored at −80 °C.

### Phenotypic screening and compound libraries

The screenings were performed by CPE-based assays in hRSV-infected Hep-2 cells. The 3244 compound library (CombinatoRx Inc.^53^) was screened in a 384-well format at a final concentration of 10 μM and was added to cells 1 h prior to inoculation with hRSV. CPE was evaluated 96 h post-infection using an ATPlite Luminescence Assay System (PerkinElmer Inc., Waltham, MA) following the manufacturer’s instructions. The cytotoxicity of compounds was assessed following the same method in absence of virus. Compounds were considered as hits if they inhibited more than 25% CPE at 10 μM, while reducing less than 30% of cell proliferation at active concentrations. The 502 compound library (Screen-Well^®^ Natural Products Library, Enzo Life Sciences Inc., Farmingdale, NY) was screened in a 96-well format at a final concentration of 0.25 μg/mL and compounds were added to cells 1.5 h post-inoculation with hRSV. Virus-induced CPE was evaluated 96 h post-infection using a CellTiter-Glo Luminescent Cell Viability Assay (Promega, Madison, WI) following the manufacturer’s instructions. The cytotoxicity of compounds was assessed following the same method in absence of virus. The percentage of CPE inhibition was calculated using the formula (signal background – signal virus-induced CPE)/(signal cytotoxicity –signal virus-induced CPE), and hits were selected if the resulting value was superior to 30%.

Cyclopamine was purchased from Logan Natural Products (Plano, TX), GDC-0449 from Selleck Chemicals (Shanghai, P.R. China), LY2940680 from Biochempartner (Shanghai, P.R. China), tomatidine from Yingxuan Pharmaceutical (Shanghai, P.R. China) and BMS-433771 from ChemPartner (Shanghai, P.R. China). The *Veratrum* steroidal alkaloids were part of the Screen-Well^®^ Natural Product Library from Enzo Life Sciences (Farmingdale, NY).

### Virus titration by focus forming assay

Dilutions of virus were adsorbed onto confluent HEp-2 cells in 24-well plates for 1.5 h at 37 °C followed by two washing steps with PBS. Cells were subsequently overlaid with 0.75% carboxymethyl cellulose in DMEM supplemented with antibiotics and 2% FBS and incubated at 37 °C for 72 h. The culture supernatant was discarded and the cells were washed 3 × 5 min with PBS before being fixed for 30 min with 4% PFA in PBS at room temperature. Cells were washed and incubated for 1 h at room temperature with a primary anti-hRSV F antibody (mouse – Fitzgerald, Acton, MA) in PBS-5% skim milk, followed by a washing step with PBS-0.02% Tween 20. Antigen-antibody complexes were revealed by incubating cells for 1 h at room temperature with a secondary goat anti-mouse HRP-conjugated antibody (Bethyl, Montgomery, TX) in PBS-5% skim milk. Cells were then washed with PBS-0.02% Tween 20 and overlaid with TrueBlue Peroxidase Substrate (KPL, Gaithersburg, MD) until blue foci appeared. The plates were then rinsed with water, dried and scanned using a Canon CanoScan 5600 F table-top scanner for focus counting and size measurement.

### Immuno-stained focus reduction assays

The anti-hRSV potency of compounds was evaluated by focus reduction assay, as previously described^54^, following the focus forming assay method and using 50 to 100 focus forming units per well. Compounds were tested during virus adsorption for 1 h at 4 °C or 37 °C to investigate inhibition of viral entry or propagation and replication, respectively, or during all stages of infection. IC_50-90-95-99_ values were determined by focus counting (viral entry) or focus size measurement (viral propagation, treatment post-adsorption) and were obtained using non-linear regression analysis using GraphPad Prism (GraphPad Software, La Jolla California USA). Compound cytotoxicity was assessed using a CellTiter-Glo Luminescent Cell Viability Assay (Promega, Madison, WI), following the manufacturer’s instruction in the conditions of the focus reduction assay.

### Selection of hRSV resistance to CPM

CPM-resistant viruses were generated by blind-passages with increasing concentration of compound. Infections were started at an MOI of 0.1 for the first round and infections were stopped after 72 h or when about 50% CPE was observed. The infection media were subsequently clarified and 100 μL of supernatant were used to inoculate cells during the next passage. A total of 8 blind passages were sequentially performed in the presence of 100 nM, 200 nM, 400 nM, 600 nM, 800 nM and 1 μM CPM. A wild-type virus passaged with DMSO only was used as control.

### Viral genome extraction, amplification and sequencing

A detailed procedure for the amplification and sequencing can be found in Supplementary Methods. Briefly, the total vRNA of each resistant supernatant was extracted using a TIANamp Virus RNA extraction Kit (Tiangen Biotech, Beijing) following the manufacturer’s instructions. The genomes were amplified using a SuperScript III One-Step RT-PCR System with Platinum Taq High Fidelity DNA Polymerase (Invitrogen, Carlsbad, CA). The fragments were checked by agarose gel electrophoresis and retrieved by gel extraction using a TIANgel Midi Purification Kit (Tiangen Biotech, Beijing), following the manufacturer’s instructions. An aliquot of each fragment was sequenced (Sangon Biotech, Shanghai), and the sequences were analyzed using the software Lasergene SeqMan Pro v.7.1 (DNASTAR, Madison, WI).

### Minigenome assay

Minigenome assays in BSR-T7/5 cells were performed as described previously^21^,^22^, using M2-1^WT^ or M2-1^R151K^ sequences. Compounds were added during transfection, and left to incubate with cells for 24 h. Briefly, BSR-T7/5 cells stably expressing the T7 RNA polymerase^55^ were transfected in triplicates in 48-well plates for 24 h in the presence of Lipofectamine 2000 (Invitrogen, Carlsbad, CA) and 125 ng of pN, 125 ng of pP, 62.5 ng of pL and 37.5 ng of pM2-1^WT^ or pM2-1^R151K^ plasmids encoding hRSV N, P, L and M2-1^WT^ or M2-1^R151K^ proteins, respectively, under control of the T7 promoter. They were co-transfected with 125 ng of a pM/Luc subgenomic minireplicon expressing the firefly luciferase gene downstream of a hRSV M/SH gene junction, as well as with 37.5 ng of a pβ-Gal plasmid coding for the β-galactosidase as a reference gene. After 24 h of transfection, cells were lysed in luciferase lysis buffer (30 mM Tris pH 7.9, 10 mM MgCl_2_, 1 mM DTT, 1% Triton X-1000, 15% glycerol). Luciferase and β-Gal activities were measured for each well as previously described (26) using a Tecan Infinite 200 PRO (Tecan Group LTD, Männedorf). Compound cytotoxicity towards BSR-T7/5 cells was assessed using a CellTiter-Glo Luminescent Cell Viability Assay (Promega, Madison, WI), following the manufacturer’s instruction in the conditions of the minigenome assay.

### Immunofluorescence microscopy

BSR-T7/5 cells were transfected as described above, using a plasmid encoding for wild-type or R151K-mutant M2-1-mCherry fusion proteins^56^ in place of the pM2-1 plasmid. The transfected cells were fixed, permeabilized and incubated with a primary mouse monoclonal anti-N antibody (AbD Serotec, clone B023) for 1 h, followed by incubation with a secondary goat anti-mouse Alexa Fluor 488 antibody for 1 h. Coverslips were mounted in Prolong Gold Antifade reagent (Invitrogen, Carlsbad, CA), observed with a Nikon TE200 microscope equipped with a CoolSNAP ES2 (Photometrics, Tucson, AZ) camera, and images were processed using the Meta-Vue (Molecular Devices, Sunnyvale, CA) and Fiji^57^ softwares.

### Immunoassays

Proteins from SDS-PAGE were transferred onto nitrocellulose membranes which were incubated with primary rabbit polyclonal anti-P^58^, -N^58^; -M2-1^26^, and mouse monoclonal anti-tubulin-α antibodies (Sigma-Aldrich, Saint-Quentin Fallavier, France) for 1 h, followed by appropriate HRP-conjugated secondary antibodies for 1 h. Immunodetections were performed using an enhanced chemiluminescence (ECL) substrate (GE Healthcare GMB, Vélizy, France). Protein levels were determined from Western blots using the GeneTools software after acquisition of the signals with a GeneGnome Digital Imager (Syngene, Frederick, MD, USA).

### Ethics Statement

The protocol for use of animals was approved by the Biological Resource Center, Singapore Animal Care and Use Committee (Protocol 070236). It was established following the Guidelines on the Care and Use of Animals for Scientific Purposes as setup by the National Advisory Committee for Laboratory Animal Research, the Animal and Birds Act (Chapter 7) and the Guide for the Care and Use of Laboratory Animals 8th Edition.

### Animal model

The antiviral efficacy of CPM was examined in the BALB/c mouse model of infection. Female BALB/c mice were obtained from the Biological Resource Center, Singapore, and used at a body weight of 16–20 g. All animals were housed in a certified, specific pathogen-free facility and were fed and watered *ad libitum*. CPM was formulated as a 0.5% hydroxypropyl methyl cellulose suspension and was administered at a dose of 30 and 100 mg/kg intraperitoneally b.i.d. for four days. BMS-433771 was dissolved in sterile water, adjusted to pH 2 to 3.5 and administered at a dose of 50 mg/kg by oral gavage b.i.d. for four days^15^. Treatments were initiated 1 h prior to hRSV inoculation. Mice were anesthetized by intraperitoneal injection of ketamine (70 mg/kg) and xylazine (20 mg/kg) and inoculated via intranasal route with 8 × 10^5^ TCID_50_ of hRSV Long Strain in 100 μL of EMEM. The control group received vehicle only. On day 4 after inoculation and 1 h after last compound administration, mice (N = 6 per group) were euthanized by CO_2_ gas asphyxiation and lungs were excised and weighed. They were homogenized (10% wt/vol) in HBSS containing 0.21 M sucrose, 25 mM HEPES, 5 mM sodium l-glutamate, 20 U/mL penicillin G, 20 μg/mL streptomycin and 0.05 μg/mL amphotericin B (GIBCO/BRL, Carlsbad, Calif.). Homogenates were frozen and thawed to release cell-associated virus and held on ice until clarification by 10 min centrifugation at 300 × g at 4 °C. The supernatants were immediately titrated for hRSV load in HEp-2 cells as described previously^15^. Final hRSV lung titers were expressed as log_10_ TCID_50_ per gram of lung. Infectious hRSV lung titers for the treatment groups, or cohorts, were calculated as mean titers and expressed as log_10_ TCID_50_ per gram of lung.

## Additional Information

**How to cite this article**: Bailly, B. *et al.* Targeting human respiratory syncytial virus transcription anti-termination factor M2-1 to inhibit *in vivo* viral replication. *Sci. Rep.* **6**, 25806; doi: 10.1038/srep25806 (2016).

## Supplementary Material

Supplementary Information
