# Supplementary material for: Targeting human respiratory syncytial virus transcription anti-termination factor M2-1 to inhibit in vivo viral replication
Source: Sci Rep. 2016 May 19;6:25806. doi: 10.1038/srep25806 (PMC4872165; doi:10.1038/srep25806)
Supplement: Supplementary Information [file srep25806-s1.pdf]

Targeting human respiratory syncytial virus transcription anti-termination factor M2-1 to inhibit *in vivo* viral replication

B. Bailly, C.-A. Richard, G. Sharma, L. Wang, L. Johansen, J. Cao, V. Pendharkar, D.-C. Sharma, M. Galloux, Y. Wang, R. Cui, G. Zou, P. Guillon, M. von Itzstein, J.-F. Eléouët, R. Altmeyer

Supplementary Methods

Sequencing of CPM-resistant hRSV supernatants

The viral RNA of each supernatant was extracted using a TIANamp Virus RNA kit, following the manufacturer's instructions (Tiangen Biotech, Beijing). The whole genome was amplified by RT-PCR using a SuperScript III One-Step RT-PCR System with Platinum Taq High Fidelity DNA Polymerase (Invitrogen, Carlsbad, CA), and six primer pairs of hRSV Long strain: RSF 1F/RSV 3231R, RSV 3131F/RSV 5798R, RSV 4572F/RSV 7620R, RSV 7575F/RSV 10759R, RSV 10591F/RSV 13161R, and RSV 12753F/RSV 15221R.

20 µl RT-PCR system:

2X Reaction Mix :	10 µl
Enzyme:	0.4 µl
Primer F/R:	0.5/0.5 µl
Template RNA:	2 µl
RNAse-free water:	6.6 µl

Reaction conditions:

50 °C:	30 min	
94 °C:	30 s	
94 °C:	15 s	40 Cycles;
55 °C:	30 s	
68 °C:	3 min	
68 °C:	5 min	

The fragments were checked by agarose gel electrophoresis and retrieved by gel extraction using a TIANgel Midi Purification Kit (Tiangen Biotech, Beijing), following the manufacturer's instructions. An aliquot of each purified fragment was sequenced (Sangon Biotech, Shanghai) using the primers presented in the following table, and the fragment assembly was performed using the Lasergene SeqMan Pro v.7.1 (DNASTAR, Madison, WI).

Primer Name	Sequence
RSV 1F	ACGCGAAAAAATGCGTACAACAAAC
RSV 536F	CTAGCAAATCAATGTCACTAGCACC
RSV 1051F	CTACACTCCATAGTCCAAATGGAGC
RSV 1576F	CTCCAGAATACAGGCATGATTCTCC
RSV 2101F	CCAGTGTAGTATTAGGCAATGCTGC
RSV 2611F	AAGACCCTATACCAAGTGATAATCCC
RSV 3131F	CCCACTCATCCAACCAAACATCC
RSV 3545F	CTTGGATGAAAGAAGCAAACTGGC
RSV 3994F	CAGCTACACGATTTGCAATCAAACCC
RSV 4572F	CATTAACATCCCACCATGCAAACC
RSV 5068F	CCCACAACAGTCAAGACTAAAAACAC
RSV 5681F	GCAAATGCAATTACCACAATCCTCG
RSV 6220F	CTTAACCAGCAAAGTGTTAGACCTC
RSV 6673F	CAGAGGATGGTACTGTGACAATGC
RSV 6983F	GTATCAAATAAAGGGGTGGACACTG
RSV 7575F	CTATCTGTAAAAATGAGAACTGGGGC
RSV 8061F	CCAGCAGACGTATTGAAGAAAACC
RSV 8484F	GCTGTGGGACAAAATGGATCCC
RSV 8917F	TCCAACAATGGACAGGATGAAGAC
RSV 9461F	AGCTATTTCACAATGAGGGGTTCTAC
RSV 10054F	CGTTTCTATCGTGAGTTTCGGTTGC
RSV 10591F	ACAGATCTCAGCAAATTCAATCAAGC
RSV 11116F	CAACATAACGGTGTATATTACCCTGC
RSV 11677F	CCTCAAGCTTTAGGGTCTGAGAGAC
RSV 12194F	CAACAAGCACTATAGCTAGTGGC
RSV 12753F	ACATTTGATGAAACCTCCCATATTCAC
RSV 13342F	AATGTAGCAGAATTTACAGTTTGCCC
RSV 13862F	CCAACCAACTTTACACTACTACTTCCC
RSV 14306F	CCGAATTGCCTGTAACAGTCAAC
RSV 14753F	CTATAGCTGGACGGAATGAAGTTTTC
RSV 1163R	CAACTTGACTTTGCTAAGAGCCATC
RSV 2202R	CAGCATATGCCTTTGCTGCATC
RSV 3231R	CTATATTGTCGATTTTTTCCAGGTGGC
RSV 4172R	CAATGACTTGGGATGATCTGAGACTTC
RSV 5798R	CACTAAGATAGCCTTTGCTAACTGC
RSV 7119R	TCATCAGAGGGGAATACTAATGGG
RSV 7620R	GATTCCTTCGTGACATATTTGCCCC
RSV 8797R	CCAACGAGGTCATACTCTTGTATGTC
RSV 9598R	CAGCATCTGTGATGTTGTTGAGC
RSV 10759R	CTCTTATATAGGGGGGTGCATGC
RSV 11864R	CTTAGCCCGTGAGGATATGTAGGTTC
RSV 13161R	CTCCAGTTTTGCTTTGCCATAACC
RSV 14066R	CCTGCTCCTTCACCTATGAATGC
RSV 15226R	ACGAGAAAAAAAGTGTCAAAAACTAATATCTCG
